# Population size estimates based on the frequency of genetically assigned parent–offspring pairs within a subsample

**DOI:** 10.1002/ece3.6365

**Published:** 2020-05-20

**Authors:** Björn Müller, Moritz Mercker, Jörg Brün

**Affiliations:** ^1^ Institute for Evolutionary Biology and Ecology University of Bonn Bonn Germany; ^2^ Bionum‐Consultants in Biological, Ecological and Biomedical Statistics Hamburg Germany

**Keywords:** density estimations in ungulates, genetic‐based capture–mark–recapture, microsatellites, *Sus scrofa*, wildlife management

## Abstract

Estimating population density as precise as possible is a key premise for managing wild animal species. This can be a challenging task if the species in question is elusive or, due to high quantities, hard to count. We present a new, mathematically derived estimator for population size, where the estimation is based solely on the frequency of genetically assigned parent–offspring pairs within a subsample of an ungulate population. By use of molecular markers like microsatellites, the number of these parent–offspring pairs can be determined. The study's aim was to clarify whether a classical capture–mark–recapture (CMR) method can be adapted or extended by this genetic element to a genetic‐based capture–mark–recapture (*g*‐CMR). We numerically validate the presented estimator (and corresponding variance estimates) and provide the R‐code for the computation of estimates of population size including confidence intervals. The presented method provides a new framework to precisely estimate population size based on the genetic analysis of a one‐time subsample. This is especially of value where traditional CMR methods or other DNA‐based (fecal or hair) capture–recapture methods fail or are too difficult to apply. The DNA source used is basically irrelevant, but in the present case the sampling of an annual hunting bag is to serve as data basis. In addition to the high quality of muscle tissue samples, hunting bags provide additional and essential information for wildlife management practices, such as age, weight, or sex. In cases where a *g*‐CMR method is ecologically and hunting‐wise appropriate, it enables a wide applicability, also through its species‐independent use.

## Graphical abstract

Estimating the population density as precise as possible is a key premise for managing wild animal species. We present a method that provides a new framework to precisely estimate population size based on the genetic analysis of a one‐time subsample. This method is species‐independent usable, and in cases where a genetic‐based capture–mark–recapture (g‐CMR) method is ecologically meaningful, it enables a wide applicability.

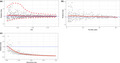

## 1 INTRODUCTION

Reliable information of population dynamics is a major pillar of estimating the impact of wildlife management actions. A key factor for population dynamics is an accurate estimation of numbers of animals living in a certain area. Counting wild animals, whether it is carnivores or herbivores, is often a demanding task, especially in nocturnal or elusive species (Bellemain, Swenson, Tallmon, Brunberg, & Taberlet, 2005; de Oliveira, do Couto, & Duarte, 2019; Eggert, Eggert, & Woodruff, 2003; Kery, Gardner, Stoeckle, Weber, & Royle, 2011). Not every method for estimating population densities is equally applicable for every species. Low‐density tending species, which concerns many carnivores (Kery et al., 2011), must be counted differently. For instance, hair traps seem to be suitable for low‐density populations (Balestrieri et al., 2010; Steyer, Simon, Kraus, Haase, & Nowak, 2013), but rather inappropriate for some high‐density ungulate species (Ebert, Huckschlag, Schulz, & Hohmann, 2009).

Collecting fecal samples is a widely used method for species monitoring and estimating population trends. They can be used in two different ways. Standard dung counts use a defecation rate in a defined area and timescale for density estimation (Eggert et al., 2003; Pfeffer et al., 2018). Alternatively, DNA analyses of the collected feces can reveal the number of different individuals, and with classical capture–mark–recapture methods (CMR), the density can be evaluated (Bellemain et al., 2005; Ebert, Knauer, Spielberger, Thiele, & Hohmann, 2012; Kery et al., 2011; Petit & Valiere, 2006). Counting fecal pellets can provide reliable results (Ferretti, Fattorini, Sforzi, & Pisani, 2016; Plhal, Kamler, & Homolka, 2014) but in some cases can lead to over‐ or underestimated densities compared to other methods (Barnes, 2001; Pfeffer et al., 2018). The choice of appropriate transects, often unavoidably high staff expenses, aggravated long‐term storage for later DNA analysis and DNA amplification, and weather‐dependent feces quality can make feces counts and genetic analyze of the fecal pellets a protracted and time‐consuming process with an uncertain outcome (Barnes, 2001; Bellemain et al., 2005; Kolodziej, Nikolov, Schulz, Theissinger, & Schulz, 2013; Soto‐Calderon et al., 2009).

Drive counts are another widely used method for estimating densities, particularly in ungulate species (Noss, Salidas, & Crespo, 2006). However, the accuracy of the results is known to be insufficient, when a high level of reliability of the estimated density is needed (Borkowski, Palmer, & Borowski, 2011). Over the last decade, camera traps became a commonly used method to estimate population density using the random encounter model (REM) by Rowcliffe, Field, Turvey, and Carbone (2008) that needs no individual recognition for density estimations (Pfeffer et al., 2018; Rowcliffe et al., 2008).

The European wild boar (*Sus scrofa*) is a species that is hard to count accurately, which can partly be explained by its complex social behavior and nocturnal activity (Briedermann, 2009; Cahil, Llimona, & Gràcia, 2003) and an exceptionally high reproductive potential compared to other ungulates of similar body size (Carranza, 1996). For more than 30 years, a Europe‐wide continuous increase of the size of wild boar populations can be observed (Baubet, Bonenfant, & Brandt, 2004; Boitani, Trapanese, Mattei, & Nonis, 1995; Cahill et al., 2003; Ferreira, Souto, Soares, & Fonseca, 2009; Keuling et al., 2013; Tsachalidis & Hadjisterkotis, 2009). This steady and partly immense population growth is basically recognizable and measurable by two factors/conditions: (a) by the annual increase in the number of shot animals in hunting bags and (b) by increased crop damages (Keuling et al., 2013). Germany has one of the highest wild boar stocks all over Europe (Keuling et al., 2013; Massei et al., 2015), and particularly, the hunting season 2017/2018 had the highest hunting bag of all times with almost 837,000 shot animals. Nevertheless, there is no clear perception of how many living wild boars there are in absolute numbers (Briedermann, 2009). But especially, the progress of African swine fever (ASF) (More et al., 2018) necessitates a reliable and accurate method to estimate their density and to adjust a proper wildlife management.

Even in Germany, the size of the remaining living populations remains mostly ambiguous due to the lack of a unified applied and exact procedure to calculate wild boar population density. Comparisons concerning the wild boar population development extending over several years or between different populations are therefore barely achievable.

The currently most common method for wild boar stocktaking is hunting bag‐based abundance calculation (Briedermann, 2009; Keuling et al., 2018). However, the annual rate of increment (between 100%–300%) or the hunting ground‐specific ecological conditions can only be guessed. Different hunting grounds are therefore difficult to compare, and the impact of current management actions is hardly assessable. Even with further attempts of improvement based on camera trap detections, drive counts, and distance sampling (Keuling et al., 2018), the comparability of estimated population sizes remains difficult without unified, standardized methods to estimate population size.

Hunting wild animals like carnivores or ungulates is not always without bias. Sport and trophy hunting, as well as sex‐ and age‐specific hunting, can affect reproduction, offspring sex ratio, body weight etc. (Milner, Nilsen, & Andreassen, 2007). Accordingly, selective harvesting can influence the age and sex composition of a hunting bag and, with that, kinship structures. Since many ungulates practice parental care, parent(s) and offspring often appear close together in their home range. This could lead to an increased proportion of parent–offspring pairs in a hunting bag, when these animals are shot together. Both circumstances could bias the results of an estimator that is based on this proportion. Regular wild boar hunting can be assumed as mostly unbiased with respect to the sex of the animals (Keuling et al., 2013; Keuling, Lauterbach, Stier, & Roth, 2010; Toigo, Servanty, Gaillard, Brandt, & Baubet, 2008), as well as unbiased toward hunting close relatives intentionally. Hunters try to avoid shooting adult females (Toigo et al., 2008) or females with piglets in general (Keuling et al., 2013), coincident with increased hunting efforts toward male yearlings and adults. A sex‐biased hunting bag toward males is not found in central Europe per se. There are both regional and annual variations, but the trend is rather toward balanced sex ratios (Keuling et al., 2013). Therefore, there seems to be no significant influence on the parent–offspring pairs in the annual hunting bag. Because of that, we expect a random and representative sample size of close relatives (like parents and offspring, full‐sibs, half‐sibs) within a sampled hunting bag.

The common data acquisition of hunting bags mainly consists of information concerning, that is, long‐term tendencies of population size, sex ratio, age composition, and sometimes state of pregnancy and litter size. But hunting bags additionally provide a whole range of genetic information, which usually remains unexploited, in particular the genetic relationships between the animals.

Genotypic analyses of fecal samples were used to develop a CMR estimator of the actual population density (Ebert et al., 2012; Ebert, Knauer, Storch, & Hohmann, 2010; Kolodziej, Schulz, et al., 2012; Kolodziej, Theissinger, Brün, Schulz, & Schulz, 2012) but require a considerable extra amount of money, work, and workforce. Some of the difficulties with fecal samples can be low DNA concentrations and low genotyping rates (e.g., Kolodziej et al., 2013). Taking fresh and high‐quality muscle tissue samples from the hunting bag could solve these problems.

With these data, kinship relations like parent–offspring pairs and kinship structures within and between populations can be revealed. Based on adaptions of classical CMR models as the Lincoln–Petersen estimator (Lincoln, 1930; Petersen, 1896; Seber, 1973; Southwood & Henderson, 2000) or the Chapman estimator (Chapman, 1951, 1954; Southwood & Henderson, 2000), these genetic relationships can be used to estimate population densities. Although no physical recapture for an already shot animal is feasible, the use of genetic information in a hunting bag, and in particular the detection and number of parent–offspring pairs, may act as a genetic recapture. Most of all, such a genetic‐based capture–mark–recapture (*g*‐CMR) model would provide a unified, reliable, and comparable method for estimating abundance and density of wild boar and other (ungulate) species.

Despite the possibility of using newer molecular methods (Genotyping by Sequencing, GBS) (Hodel et al., 2016; Sonah et al., 2013), microsatellites (Simple Sequence Repeats, SSR) still belong to the most frequently used genetic markers for reliable individual identification (reviewed in Hodel et al., 2016; Selkoe & Toonen, 2006). Microsatellites allow for accurate estimations of parent–offspring as well as full‐sib and half‐sib relationships (Christie, 2010; Costa et al., 2012; Putnova, Knoll, Dvorak, & Dvorak, 2003).

In principle, the number of parent–offspring pairs can be determined from all available DNA sources, for example, muscle tissue, hair samples, or feces. Furthermore, it should be possible to estimate the size of a population based on the number of these parent–offspring pairs within a subsample: The larger the population is (while keeping the size of a subsample constant), the lower is the chance that parents and offspring appear together within the subsample. Thus, the estimated population size should increase with a decreasing number of parent–offspring pairs within the subsample. We thus aimed to create a protocol and R‐script that allows for estimating and simulating population densities together with the opportunity to validate the results. In order to estimate a possible influence of the above‐mentioned hunting bias on a hunting bag, different scenarios should be considered. Below, we mathematically derive and numerically validate corresponding population estimators.

## 2 DERIVATION OF THE ESTIMATORS

Let *N* be the true size of a closed population *N*, where *N_F_* is the number of adult females, *N_M_* is the number of adult males, and *N_J_* is the number of juveniles, thus *N = N_F_ + N_M_ + N_J_*. Let further *n* be the size of a subsample of *N*, where *n_F_* is the number of adult females in the subsample, *n_M_* is the number of adult males in the subsample, and *n_J_* is the number of juveniles, thus *n = n_M_ + n_F_ + n_J_*. Finally, *m_FC_* is the number of father–offspring pairs in the subsample, and *m_MC_* is the number of mother–offspring pairs.

The average chance of any juvenile being in the same subsample as their parent depends on the relative portion of all *N_M_* adult males (*N_F_* females) in this subsample: If for example 50% of the adult males are within the subsample, for each juvenile in the subsample the average chance that its father is also within the subsample is 0.5. It follows for the expected values that

$$E\left(m_{\mathrm{FC}}/n_{J}\right)=E\left(n_{M}/N_{M}\right)=E\left(n_{M}\right)/N_{M}$$

respectively

$$E\left(m_{\mathrm{MC}}/n_{J}\right)=E\left(n_{F}/N_{F}\right)=E\left(n_{F}\right)/N_{F}$$

Assuming that there is always at least one mother–offspring respectively one father–offspring pair within the subsample (i.e., *m_FC_ *> 0 and* m_MC_ *> 0, otherwise the population size cannot be estimated), simple rewriting of the equation leads to

$$N_{M}=E\left(\frac{n_{M}*n_{J}}{m_{\mathrm{FC}}}\right)$$

respectively

$$N_{F}=E\left(\frac{n_{F}*n_{J}}{m_{\mathrm{MC}}}\right)$$

Leading to the corresponding estimators

$${\hat{N}}_{M}=\frac{n_{M}*n_{J}}{m_{\mathrm{FC}}}$$

respectively

$${\hat{N}}_{F}=\frac{n_{F}*n_{J}}{m_{\mathrm{MC}}}$$

Thus, an estimator for all adult animals $N_{A}=N_{M}+N_{F}$ is given by

$${\hat{N}}_{A}=\frac{n_{F}*n_{J}}{m_{\mathrm{MC}}}+\frac{n_{M}*n_{J}}{m_{\mathrm{FC}}}=n_{J}*\left(\frac{n_{F}}{m_{\mathrm{MC}}}+\frac{n_{M}}{m_{\mathrm{FC}}}\right).$$

which is strongly related to the Lincoln–Petersen estimator (Lincoln, 1930; Petersen, 1896) that is frequently used in the capture–mark–recapture analysis. In our derived estimator, we require randomly chosen males (females, juveniles) from the entire population of males (females, juveniles), but the proportion of males versus females versus juveniles in the subsample does not have to be representative. This is an important issue, especially when the catching probability of animals depends on sex or age.

However, if the true number of juveniles *N_J_* has to be estimated as well, an estimator for the average number of juveniles per adult is required, in the following denoted by *Ĵ*. If such an estimator is given, *N* can be estimated via

$$\hat{N}={\hat{N}}_{A}+\hat{J}*{\hat{N}}_{A}$$

If the proportion of males versus females versus juveniles in the subsample can be assumed to be representative for the entire population (i.e., the catching probability does not depend on sex or age), a straight forward definition of *Ĵ* is given by

$$\hat{J}=n_{J}/\left(n_{M}+n_{F}\right)$$

thus

$$\hat{N}={\hat{N}}_{A}+\frac{n_{J}}{n_{M}+n_{F}}*{\hat{N}}_{A}$$

$$=n_{J}*\left(\frac{n_{F}}{m_{\mathrm{MC}}}+\frac{n_{M}}{m_{\mathrm{FC}}}\right)*\frac{{\left(n_{J}\right)}^{2}}{n_{M}+n_{F}}*\left(\frac{n_{F}}{m_{\mathrm{MC}}}+\frac{n_{M}}{m_{\mathrm{FC}}}\right)$$

$$=n_{J}*\left(\frac{n_{F}}{m_{\mathrm{MC}}}+\frac{n_{M}}{m_{\mathrm{FC}}}\right)*\left(1+\frac{n_{J}}{\left(n_{M}+n_{F}\right)}\right)$$

## 3 VARIANCE ESTIMATION

In the following, we estimate the variance of the estimators by using bootstrap methods, which have been shown to yield reliable variance estimations if the analytical calculations are complex (Canty, Davison, Hinkley, & Ventura, 2006; Davison & Hinkley, 1997; Efron & Tibshirani, 1994). Especially, for each subsample of size *n*, we create *j* = 1,..., *n*_boot_ random resamples with replacement (of size *n*) and calculate for each resample *j* the corresponding estimator value; the final variance is then calculated based on the quantiles of the *n*_boot_ estimator values.

## 4 VALIDATION OF THE ESTIMATORS

In order to validate the estimators, it is worth mentioning that for a given population *N*, the estimators ${\hat{N}}_{M}$ , ${\hat{N}}_{F}$ , ${\hat{N}}_{A}$ , and $\hat{N}$ differ on average only by one (not necessarily even) number, so that the relative bias (i.e., the population estimate normalized by the true population size, $\hat{N}$ /*N*) should show an identical behavior for all estimators. It is thus sufficient to numerically validate one of them, for example, $\hat{N}$ .

**FIGURE 1:**
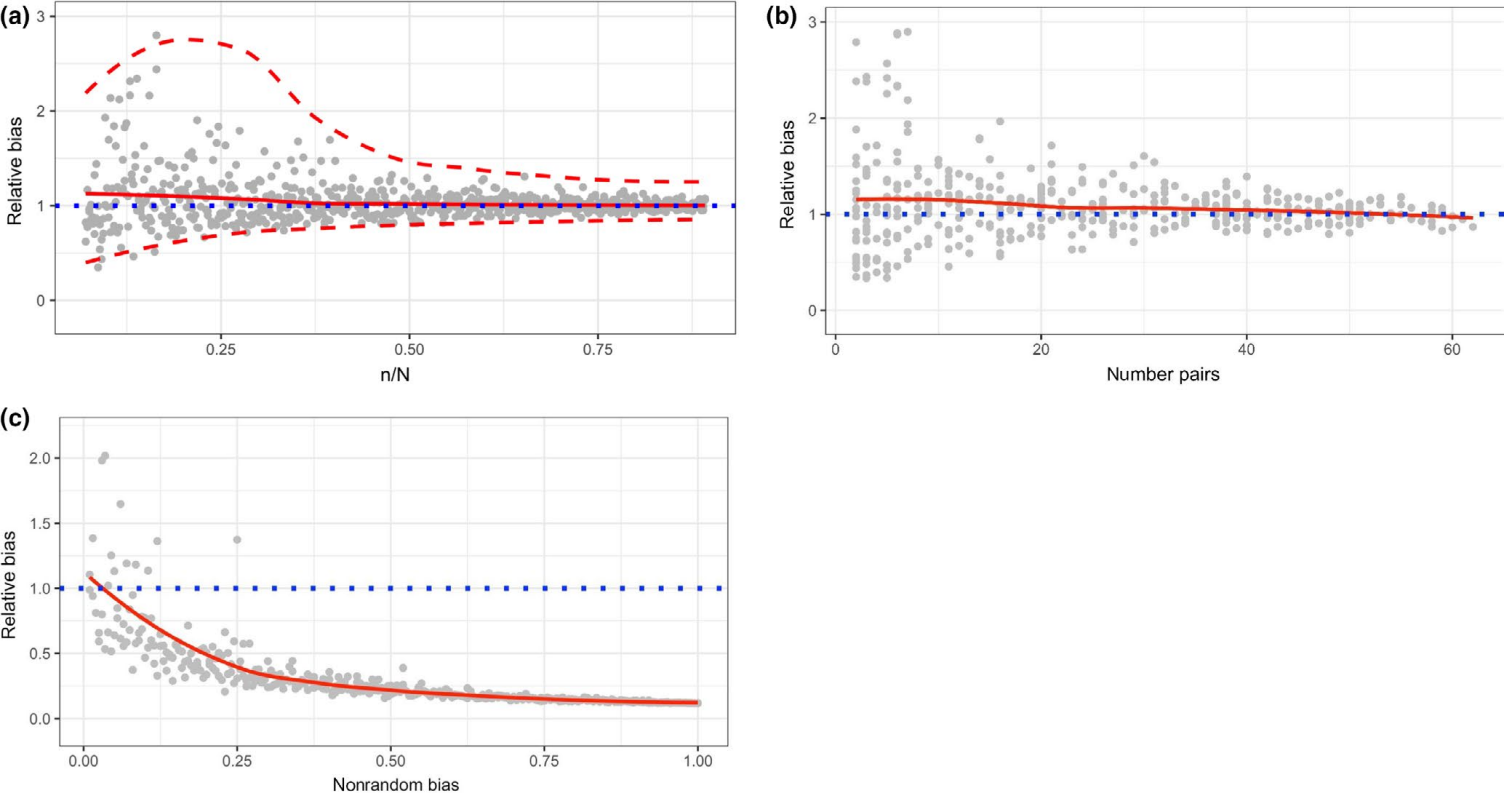
(a) Numerical (Monte Carlo) validation of the relative bias and the coverage probability of the estimator. Especially, the influence of different relative subsample sizes *n*/*N* is investigated. Gray dots: Relative bias estimated for a single subsample; red continuous line: average smooth of the single estimates; red dashed lines: average smooth of the upper and lower 95% confidence limits as calculated based on 200 bootstrap resamples; blue dotted line: unbiased value at 1. Smooths are based on LOESS smoothing, the total size of the virtual population is *N* = 400. (b) The same simulation framework and legend as in (a), but estimator bias is plotted against the total number of parent*–*offspring pairs per sample. (c) The same simulation framework and legend as in (a, b) (except a fixed subsample size of *n* = 50) but “nonrandom bias” in the hunting bag has been additionally introduced and plotted against estimator bias. Especially, a nonrandom bias = 0 means that there is no per se increased chance that parents and offspring occur together in the hunting bag; a nonrandom bias = 0.5 means a 50% probability for an offspring being shoot together with either its mother or father; a nonrandom bias = 1 means a 100% probability for an offspring being shoot together with its mother and father

The validation of the bias and coverage probability has been done based on Monte Carlo methods. Specifically, we first created a virtual population of 100 males, 100 females, and 200 offspring (thus *N* = 400), where the number of offspring per mother has been randomly generated based on a Poisson distribution with expected value *λ *= 2, and the father has been randomly assigned for each offspring. In a second step, we randomly selected a subsample of size *n*, and based on this subsample, we finally estimated the population size as well as 95% confidence intervals, and the latter based on 200 bootstrap resamples. The second step has been repeated for *n* = 20, 21, 22,…, 360; thus, the relative subsample size *n*/*N* ranged between 0.05 and 0.9. The corresponding results are shown in Figure 1a. It appears that the estimator is only slightly positively biased for small values *n/N* and appears to be unbiased with increasing *n*/*N*. However, for *n*/*N* = 0.05 the relative bias is still <10%. Additionally, the calculated confidence intervals show a reasonable experimental coverage, even for small values of *n*/*N*.

To estimate a possible hunting distortion on a hunting bag, we simulated the following two scenarios: (a) the influence of random harvest efforts on parent–offspring pairs staying together in their home range and (b) the influence of harvest efforts on parent–offspring pairs when not random, but purposefully caused by hunt. In Figure 1b, the bias is plotted against the number of parent–offspring pairs in the hunting bag, demonstrating that the estimator works well (respectively is only slightly positively biased) if only a few pairs are available. Finally, Figure 1c demonstrates that when parent–offspring pairs are harvested together (e.g., by hunters’ purpose), the estimator shows a strong negative bias, revealing the importance of considering only populations (respectively, hunting techniques and conventions) where parent–offspring pairs do not per se have a higher chance to occur together in the same hunting bag.

## 5 DISCUSSION

We derived and validated an estimator for the population size based on the number of parent–offspring pairs in a subsample. Based on Monte Carlo simulations, we demonstrated that the estimator is only slightly biased when relative subsample sizes are small and asymptotically unbiased when the relative subsample size increases. Calculated confidence limits showed a reasonable coverage probability. Finally, we provided the R‐code for the calculations as well as some examples in order to make this method easily available for any researcher (see Appendix [Link], [Link], [Link]). The presented approach allows an accurate estimate of population size based on the genetic analysis of a subsample and thus offers a simple and attractive alternative to frequently used capture–mark–recapture methods, especially if the latter are difficult or impossible to apply.

An appropriate wildlife management for wild boar or any other wild animal species can only be implemented when it is based on reliable and current data. For more than 30 years, people recognized the importance of having information concerning the annual growth rate and the relationship between the living stock and the hunting bag to calculate population trends as precise as possible (Briedermann, 1986, 2009). However, calculating reliable trends needs long‐term data (Acevedo et al., 2007) but such data are only of limited benefit for calculating current population density (Keuling et al., 2018). Since the annual population density and hunting bag can strongly fluctuate, it is essential to update the actual density every year (Briedermann, 2009). The technical advances, particularly in molecular genetic protocols combined with the here presented *g*‐CMR, shall help to take an important step forward to accomplish this objective.

The usage of classical capture–mark–recapture (CMR) provides reliable and high‐grade results concerning animal densities. However, there are some disadvantages making this method outdated. Capturing, for example, ungulates is time‐consuming and, in some countries, demands extensive administration. In high‐density populations, where the need for reduction is a main management purpose, releasing captured animal seems paradox (Keuling et al., 2018). Simultaneously, modern spatial capture–recapture models (SCR) are replacing traditional CMR in wildlife monitoring (Jimenez, Higuero, Charre‐Medellin, & Acevedo, 2017; Kery et al., 2011). Using DNA data as a component of recapture instead of physical recapture for estimating population densities in a CMR or SCR environment also provides results with high quality and accuracy (Ebert et al., 2012; Kery et al., 2011). Compared to DNA‐based sampling methods like feces sampling (Bellemain et al., 2005; Ebert et al., 2012; Kolodziej et al., 2013), genotyping of the hunting bag requires less time and sampling effort and provides a steady and faster genotyping success due to superior DNA quality of muscle tissue. Additionally, this method gives a surplus of information regarding the population. For any sampled individual, it is possible to collect information concerning the weight, approximately age, the general body condition etc. For females, period of gestation and number of embryos can be calculated. Basically, the sex can be determined by molecular methods (e.g., Fontanesi, Scotti, & Russo, 2008), but is normally not required when sampling a hunting bag. Combining all available information results in an improved insight in the investigated population. Further statistical analyses could reveal different preferences of individuals and age classes (piglets, subadult and adult) in terms of mate choice preferences or the individual reproductive success. All that appears helpful for further improvements in managing populations. Knowing the density of a population as precisely as possible is one part of wildlife management. To know the impact, and its reasons, of the different population members in the population growth is another part.

The estimation procedure is robust to certain deviations from random sampling and smaller sample sizes. A deviation of less than 10% of the real population size appears to be tolerable. In that case, the estimator rather tends to overestimate the real population size, but as the magnitude of the error can be estimated, it can be considered for future management actions. However, too small sample sizes or subsamples appears rather unrealistic for many European ungulates. But when investigating, for example, small carnivore populations (Kery et al., 2011), the results of the here developed estimator must be treat with caution.

For species with a hunting regime that is focused on specific or purposefully harvest on parent–offspring pairs, the estimator seems to become inaccurate and inappropriate and tends to underestimate the real population size. It is therefore recommended not to rely on one method alone, but to always use at least two different methods for mutual validation. Combining the here presented estimator with, for example, a noninvasive sampling and density‐estimating method (e.g., Ebert et al., 2012; Kery et al., 2011; Mollet, Kery, Gardner, Pasinelli, & Royle, 2015), could provide more than promising results, allowing both long‐term and sustainable wildlife management.

Both CMR models, Lincoln–Petersen and Chapman estimator, assume that the investigated population in question is closed and without any migration. This also applies to our model. Although real populations are always affected by both immigration and emigration, as a first approach it makes sense to treat populations as closed, especially as reliable data on migration are hard to come by. In case of wild boar, this error is rather small since especially females are faithful to their habitats and have comparatively small home ranges (Keuling, Stier, & Roth, 2008, 2009). However, this may not be true for other species, both ungulates and carnivores. Further studies may aim to extend the present model with data on migration.

## CONFLICT OF INTEREST

None declared.

## AUTHOR CONTRIBUTION

**Björn Müller:** Conceptualization (equal); Formal analysis (supporting); Methodology (equal); Project administration (lead); Writing‐original draft (lead); Writing‐review & editing (lead). **Moritz Mercker:** Conceptualization (equal); Formal analysis (lead); Methodology (equal); Software (lead); Writing‐original draft (supporting); Writing‐review & editing (supporting). **Jörg Brün:** Conceptualization (equal); Methodology (equal); Supervision (lead); Writing‐original draft (supporting); Writing‐review & editing (supporting).

## Supporting information

Appendix S1

Appendix S2

Appendix S3

## Data Availability

All generated R‐scripts will be available as online electronic appendices or will be downloadable on our institute website.
