# Supplementary material for: Population size estimates based on the frequency of genetically assigned parent–offspring pairs within a subsample
Source: Ecol Evol. 2020 May 20;10(13):6356–63. doi: 10.1002/ece3.6365 (PMC7381586; doi:10.1002/ece3.6365)
Supplement: Supplementary file 1 — Appendix S1 [file ECE3-10-6356-s001.pdf]

```
#-------------------------------------------------------------------------------
#***** LIBRARIES AND WORKING DIRECTORY  ****************************************
#-------------------------------------------------------------------------------


#The ESTIMATE() function needs the three inputs "n_data", "n_boot" and "minus_sub".


#n_data

  #contains the data of the subsample including all individuals and parent-child
  relationships.

  #The variable "Sex" has to have 3 levels: "W" for Female, "M" for Male, "K" for
  offspring/juvenile.
  #The variable "ID" has to be unique for every individual
  #The variable "Mother" has to specify for each offspring/juvenile the ID of the mother if
  it is in the subsamle, NA otherwise
  #The variable "Father" has to specify for each offspring/juvenile the ID of the father if
  it is in the subsamle, NA otherwise

  #ALL THREE VARIABLES HAVE TO BE OF TYPE "character"

  #Thus, the data frame should look like this:

  # Sex   ID  Mother Father
  # W     55  NA     NA
  # W     76  NA     NA
  # M     172 NA     NA
  # M     256 NA     NA
  # K     339 172    NA
  # K     356 NA     76
  # K     379 NA     NA
  # ....

#n_boot

  #This variable specifies the number of boot-resamples. Ofthe recommended is the number of
  n_boot=2000, however, this will take some minutes to calculate


#minus_sub

  #if minus_sub=1, it means that the individuals of the subsample are substracted from the
  population estimate. This e.g. apples if the animals of the subsample have been killed,
  and the estimate of the population should apply only to all living animals.
  #Otherwise: minus_sub = 0.


ESTIMATE <- function(n_data,n_boot,minus_sub)
{

cat("Calculating...\n")

estimator <-  function(n_data,minus_sub)
{

  n_F <-  nrow(n_data[n_data$Sex=="W",])
  n_M <-  nrow(n_data[n_data$Sex=="M",])
  n_A <- n_F + n_M
  n_J  <- nrow(n_data[n_data$Sex=="K",])

  n_kinder <- 0
  n_Mother_kinder <- 0
  n_Father_kinder <- 0

  ####################
  #Sibling Analysis
  idat <- n_data[n_data$Sex=="K",]
  num <- as.numeric()
```

```
  for(zr in unique(idat$Mother))
  {
    num <- c(num,nrow(idat[idat$Mother==zr,]))

  }

  for(u in unique(n_data[n_data$Sex=="K",]$ID))
  {
    Mother <- n_data[n_data$ID==u,]$Mother
    Father <- n_data[n_data$ID==u,]$Father
    n_kinder <- n_kinder+ nrow(n_data[n_data$ID%in%c(Mother,Father),])
    n_Mother_kinder <- n_Mother_kinder+ nrow(n_data[n_data$ID%in%c(Mother),])
    n_Father_kinder <- n_Father_kinder+ nrow(n_data[n_data$ID%in%c(Father),])
  }

  n_Cmother <- n_Mother_kinder
  n_Cfather <- n_Father_kinder




N_F <- NA
N_M <- NA
N_A <- NA
N_J <- NA
N <-   NA
if( (n_Cmother>0)&(n_F>0)&(n_J>0))
{
  N_F <-    (n_F*n_J/n_Cmother)
}

if( (n_Cfather>0)&(n_M>0)&(n_J>0) )
{
  N_M <- (n_M*n_J/n_Cfather)
}

if((!is.na(N_F))&(!is.na(N_M)))
{
 N_A <- N_F + N_M
}

if(!is.na(N_A)&(n_J>0))
{
  N_J <- N_A*( (n_J)/(n_M + n_F) )
}

if((is.na(N_A))&(!is.na(N_M)))
{
  N_J <- N_M*( (n_J)/(n_M) )
}

if((is.na(N_A))&(!is.na(N_F)))
{
  N_J <- N_F*( (n_J)/(n_F) )
}
if((!is.na(N_A))&(!is.na(N_J)))
{
N <-  N_A + N_J
}

if(minus_sub==1)
{
  try({
    N_F <-  N_F - n_F
  })
  try({
    N_M <-  N_M - n_M
  })
  try({
    N_A <-  N_A - n_A
  })
```

```
  try({
    N_J <-  N_J - n_J
  })
  try({
    N <-  N - nrow(n_data)
  })
}

dat <- data.frame(estimates = c(N_F,N_M,N_A,N_J,N))
rownames(dat) <- c("N_Females","N_Males","N_Adults","N_Juveniles","N_total")
return(dat)
}


boot_estimator <-  function(n_data,minus_sub)
{

  n_F <-  nrow(n_data[n_data$Sex=="W",])
  n_M <-  nrow(n_data[n_data$Sex=="M",])
  n_A <- n_F + n_M
  n_J  <- nrow(n_data[n_data$Sex=="K",])

  n_kinder <- 0
  n_Mother_kinder <- 0
  n_Father_kinder <- 0

  ####################
  #Sibling Analysis
  idat <- n_data[n_data$Sex=="K",]
  num <- as.numeric()
  for(zr in (idat$Mother))
  {
    num <- c(num,nrow(idat[idat$Mother==zr,]))

  }

  for(u in (n_data[n_data$Sex=="K",]$ID))
  {
    Mother <- n_data[n_data$ID==u,]$Mother
    Father <- n_data[n_data$ID==u,]$Father
    n_kinder <- n_kinder+ nrow(n_data[n_data$ID%in%c(Mother,Father),])
    n_Mother_kinder <- n_Mother_kinder+ nrow(n_data[n_data$ID%in%c(Mother),])
    n_Father_kinder <- n_Father_kinder+ nrow(n_data[n_data$ID%in%c(Father),])
  }

  n_Cmother <- n_Mother_kinder
  n_Cfather <- n_Father_kinder



    XXX <- estimator(n_data,minus_sub)



  N_F <- NA
  N_M <- NA
  N_A <- NA
  N_J <- NA
  N <-   NA
  if( (n_Cmother>0)&(n_F>0)&(n_J>0)&(!is.na(XXX[1,1])))
  {
    N_F <-   (n_F*n_J/n_Cmother)
  }

  if( (n_Cfather>0)&(n_M>0)&(n_J>0)&(!is.na(XXX[2,1])) )
  {
    N_M <- (n_M*n_J/n_Cfather)
  }

  if((!is.na(N_F))&(!is.na(N_M))&(!is.na(XXX[3,1])))
  {
```

```
    N_A <- N_F + N_M
  }

  if(!is.na(N_A)&(n_J>0)&(!is.na(XXX[4,1])))
  {
    N_J <- N_A*( (n_J)/(n_M + n_F) )
  }

  if(is.na(N_A)&(!is.na(N_F))&(n_J>0)&(!is.na(XXX[4,1])))
  {
    N_J <- N_F*( (n_J)/(n_F) )
  }

  if(is.na(N_A)&(!is.na(N_M))&(n_J>0)&(!is.na(XXX[4,1])))
  {
    N_J <- N_M*( (n_J)/(n_M) )
  }

  if((!is.na(N_A))&(!is.na(N_J))&(!is.na(XXX[5,1])))
  {
    N <-  N_A + N_J
  }

  dat <- data.frame(estimates = c(N_F,N_M,N_A,N_J,N))
  rownames(dat) <- c("N_Females","N_Males","N_Adults","N_Juveniles","N_total")
  return(dat)
}

boot_variance <- function(xdata,n_boot,minus_sub)
{
  #n_boot <-  100
  #xdata <-  n_data
  NN_hat <- as.numeric()
  NN_hat2 <- as.numeric()
  NN_hat3 <- as.numeric()
  NN_hat4 <- as.numeric()
  NN_hat5 <- as.numeric()

  XXX <- estimator(xdata,minus_sub)


 for(i in 1:n_boot)
  {
    #i <-  1
    n_data <- xdata[ sample( c(1:nrow(xdata)),size=nrow(xdata),replace=T), ]
    res <- boot_estimator(n_data,minus_sub)
    NN_hat <- c(NN_hat,res[1,])
    NN_hat2 <- c(NN_hat2,res[2,])
    NN_hat3 <- c(NN_hat3,res[3,])
    NN_hat4 <- c(NN_hat4,res[4,])
    NN_hat5 <- c(NN_hat5,res[5,])
  }


  d1 <- as.numeric(quantile(NN_hat,prob=c(0.05),na.rm=T))
  d2 <- as.numeric(quantile(NN_hat2,prob=c(0.05),na.rm=T))
  d3 <- as.numeric(quantile(NN_hat3,prob=c(0.05),na.rm=T))
  d4 <- as.numeric(quantile(NN_hat4,prob=c(0.05),na.rm=T))
  d5 <- as.numeric(quantile(NN_hat5,prob=c(0.05),na.rm=T))

  u1 <- as.numeric(quantile(NN_hat,prob=c(0.95),na.rm=T))
  u2 <- as.numeric(quantile(NN_hat2,prob=c(0.95),na.rm=T))
  u3 <- as.numeric(quantile(NN_hat3,prob=c(0.95),na.rm=T))
  u4 <- as.numeric(quantile(NN_hat4,prob=c(0.95),na.rm=T))
  u5 <- as.numeric(quantile(NN_hat5,prob=c(0.95),na.rm=T))

  res <-  list()
  res[[1]] <- c(d1,d2,d3,d4,d5)
  res[[2]] <-  c(u1,u2,u3,u4,u5)
  return(res)
}
```

```r
DAT <-  estimator(n_data,minus_sub)

DAT <-  data.frame(estimates=as.numeric(t(DAT)))
rownames(DAT) <- c("N_Females","N_Males","N_Adults","N_Juveniles","N_total")

cat("Bootstrap-resampling...\n")
VAR <- boot_variance(n_data,n_boot,minus_sub)


DAT$CI_down <- VAR[[1]]
DAT$CI_up <- VAR[[2]]
DAT

cat("\n...done\n")
cat("\n RESULTS: \n")

return(DAT)

}




######### APPLICATION ##############


n_data <- read.table("Example Input File.txt",header=T)
head(n_data)

n_data$Sex <- as.character(n_data$Sex)
n_data$ID <- as.character(n_data$ID)
n_data$Mother <- as.character(n_data$Mother)
n_data$Father <- as.character(n_data$Father)

ESTIMATE(n_data,n_boot=100,minus_sub=0)
```
