# Supplementary material for: Population size estimates based on the frequency of genetically assigned parent–offspring pairs within a subsample
Source: Ecol Evol. 2020 May 20;10(13):6356–63. doi: 10.1002/ece3.6365 (PMC7381586; doi:10.1002/ece3.6365)
Supplement: Supplementary file 2 — Appendix S2 [file ECE3-10-6356-s002.pdf]

```r
#*******************************************************************************
# Population Estimator
#*******************************************************************************
#
# (c) Moritz Mercker / BIONUM  2018
#
#-------------------------------------------------------------------------------

#-------------------------------------------------------------------------------
#***** Delete Working Memory           ****************************************
#-------------------------------------------------------------------------------

rm(list=ls(all=TRUE))

#-------------------------------------------------------------------------------
#***** LIBRARIES UND WORKING DIRECTORY  ****************************************
#-------------------------------------------------------------------------------


setwd("/C:/Users/Public/Documents/")


source("ESTIMATE_function.R")

N <- 100 # Number of Females; the Numbers are Freely Editable
M <- 100 # Number of Males; the Numbers are Freely Editable
K <- 2 # Average Number of Children per Female; the Numbers are Freely Editable


data <- data.frame(Sex=
c(rep("W",N),rep("M",M)),ID=c(1:(N+M)),Mother=rep(NA,N+M),Father=rep(NA,N+M))


m_data <- data[data$Sex=="M",]
w_data <- data[data$Sex=="W",]

for(i in unique(w_data$ID))
{
  N_kind <- rpois(lambda=K,n=1)

  #Full Siblings:
  Mother <- sample(unique(w_data$ID),size=1)
  Father <- sample(unique(m_data$ID),size=1)
  ################

  for(j in 1:N_kind)
  {
    newline <- data.frame(Sex="K",ID=(nrow(data)+1),Mother=Mother,Father=Father)
    data <- rbind(data,newline)
  }


}

N_true <- nrow(data)
K_true <- nrow(data[data$Sex=="K",])
W_true <- nrow(data[data$Sex=="W",])
M_true <- nrow(data[data$Sex=="M",])

#### Draw Sample:

##### TEST
#n_data <- data[sample(nrow(data),size=100,replace=F), ]
#ESTIMATE(n_data,n_boot=100)
########


NN <- as.numeric()
mean <- as.numeric()
mean2 <- as.numeric()
```

```r
uup4 <- as.numeric()
ddo4 <- as.numeric()
suup4 <- as.numeric()
sddo4 <- as.numeric()
n <- 20


Dat1 <- data.frame()
Dat2 <- data.frame()
Dat3 <- data.frame()
Dat4 <- data.frame()

points <- data.frame()


for(tt in 20:400)
{

n <-  tt #Sample Size
m <-  2 #Resamples
#n <-  100

cat("Rel. Stichprobengroesse: ",n/N_true,"\n")


for(zz in 1:m)
{

test <-  0
while(test==0)
{
test <-  0

#TEST
#n <-  100

n_data <- data[sample(nrow(data),size=n,replace=F), ]
Res <- ESTIMATE(n_data,n_boot=150,minus_sub=0)
 if(!is.na(Res[5,1]))
 {
  test <- 1
 }

}

newline <- data.frame(value=Res[5,1]/N_true,down=Res[5,2]/N_true, up = Res[5,3]/N_true,n=n)
points <-  rbind(points,newline)

}


}

saveRDS(points,"./temp_files/points_rel_0.RDS")


library(ggplot2)

points <-  readRDS("./temp_files/points_rel_0.RDS")
line <- ggplot(points,aes(n/N_true,value))
line +  labs(x="n/N",y="relative bias")  + geom_point(aes(n/N_true,value),colour="grey") +
geom_smooth(method="loess", colour="red",se = F) +
geom_smooth(aes(n/N_true,down),method="loess", linetype="dashed", colour="red",se = F)  +
geom_smooth(aes(n/N_true,up),method="loess", linetype="dashed", colour="red",se = F) +
theme_bw() + coord_cartesian(ylim=c(-0.2, 3)) + geom_hline(yintercept =
1,linetype="dotted",color="blue",size=1.3)
ggsave("Plot.jpeg",width=15,height=8,units="cm",dpi=1000)
```
