# Supplementary material for: Population size estimates based on the frequency of genetically assigned parent–offspring pairs within a subsample
Source: Ecol Evol. 2020 May 20;10(13):6356–63. doi: 10.1002/ece3.6365 (PMC7381586; doi:10.1002/ece3.6365)
Supplement: Supplementary file 3 — Appendix S3 [file ECE3-10-6356-s003.pdf]

```
#***************************************************************************
# Population Estimator
#***************************************************************************
#
# (c) Moritz Mercker / BIONUM  2018
#
#---------------------------------------------------------------------------

#---------------------------------------------------------------------------
#***** Delete Working Memory            ***********************************
#---------------------------------------------------------------------------

rm(list=ls(all=TRUE))

#---------------------------------------------------------------------------
#***** LIBRARIES AND WORKING DIRECTORY  ***********************************
#---------------------------------------------------------------------------


setwd("/C:/Users/Public/Documents/") # The shown working directory is just an example. To
view the currently used working directory, enter "getwd()" in the R console


#############################################
######## CREATE A VIRTUAL POPULATION ###########
#############################################



N <- 100 # Number of Females; the Numbers are Freely Editable
M <- 100 # Number of Males; the Numbers are Freely Editable
K <- 2 # Average Number of Children per Female; the Numbers are Freely Editable


data <- data.frame(Sex=
c(rep("W",N),rep("M",M)),ID=c(1:(N+M)),Mother=rep(NA,N+M),Father=rep(NA,N+M))


m_data <- data[data$Sex=="M",]
w_data <- data[data$Sex=="W",]

for(i in unique(w_data$ID))
{
  N_kind <- rpois(lambda=K,n=1)

  #Full Siblings:
  Mother <- sample(unique(w_data$ID),size=1)
  Father <- sample(unique(m_data$ID),size=1)
  ################

  for(j in 1:N_kind)
  {
    newline <- data.frame(Sex="K",ID=(nrow(data)+1),Mother=Mother,Father=Father)
    data <- rbind(data,newline)
  }


}

N_true <- nrow(data)
K_true <- nrow(data[data$Sex=="K",])
W_true <- nrow(data[data$Sex=="W",])
M_true <- nrow(data[data$Sex=="M",])



#############################################
######## TAKE A RANDOM SUBSAMPLE      ###########
#############################################
```

```
size <-  100 # The Size of the Subsample is Freely Editable

n_data <- data[sample(nrow(data),size=size,replace=F), ]


################################################
######## ESTIMATE THE POPULATION SIZE ###########
################################################

source("ESTIMATE_function.R")

#True Number:
cat("True number:", nrow(data), "\n")

#Estimated Number:
ESTIMATE(n_data,n_boot=100,minus_sub=0) # n_boot=x Specifies the Number of Boot-Resamples;
if minus_sub=1, it means that the individuals of the subsample are substracted from the
population estimate; otherwise: minus_sub=0
```
